# Point of Care Ultrasound Diagnosis of a Massive Thoracoabdominal Aortic Aneurysm

**DOI:** 10.7759/cureus.1611

**Published:** 2017-08-26

**Authors:** Allison M Yee, Cyrus V Etebari, Srikar Adhikari, Richard Amini

**Affiliations:** 1 College of Medicine, University of Arizona; 2 Department of Emergency Medicine, University of Arizona

**Keywords:** abdominal aortic aneurysm, thoracic aortic aneurysm, vascular surgery

## Abstract

This report highlights an atypical presentation of extensive thoracoabdominal aortic aneurysm with intramural hematoma and transient paralysis of the lower extremities. Clinical suspicion for aortic pathology prompted a point of care ultrasound of the heart and aorta, which demonstrated a thoracic and abdominal aortic aneurysm with intraluminal pathology. Consultation and transfer to a tertiary care facility was based solely on the emergency physician’s ultrasound. Subsequent computed tomography (CT) imaging confirmed the ultrasound findings and discovered a left common iliac artery thrombosis consistent with the patient’s presentation. Point of care ultrasound can help clinicians diagnose aortic pathology and direct patient care efficiently and effectively.

## Introduction

Non-dissecting thoracic and abdominal aortic aneurysms (AAA) may present as intense abdominal pain with referred back pain. An AAA is defined as an aorta larger than 3 cm in diameter or a dilation of the aorta of greater than 50% [1]. Although the risk factors for AAA include tobacco use, male gender, and a history of hypertension; a diastolic blood pressure above 100 mmHg is the most consistent risk factor for the formation of an aneurysm [2]. In recent years, the worldwide incidence for AAA has increased to as high as 11% [1]. Successful identification of an aortic aneurysm is critical in preventing the formation of an aortic dissection, which accounts for 80% of mortality in patients with aortic aneurysms [1]. Aortic aneurysms greater than or equal to 7 cm in diameter have a 20-40% annual risk of rupture; as a result, these high-risk patients are offered surgical repair [3]. The literature describes patients with AAA and dissections presenting with acute onset lower extremity paralysis due to spinal cord ischemia, but to our knowledge, there are no cases describing lower extremity paralysis due to aneurysm and thrombosis of the iliac arteries [4-8].

## Case presentation

A 55-year-old male with a past medical history of chronic back pain, presented to the emergency department (ED) with acute-onset chest pain, back pain and accompanying temporary lower extremity paralysis. The patient states that the chest pain began while driving. He described the pain as a sharp, wrenching, 10 out of 10 pain, that started in his chest and radiated to his back in between his shoulder blades. The patient described an accompanying acute tingling sensation down his body that led to the temporary inability to use both of his lower extremities. Unable to brake, the patient steered his vehicle to a natural stop and then called for an ambulance. At the time of evaluation in the ED, the patient had regained the use of his right lower extremity, and was slowly regaining function of his left lower extremity. The patient also reported decreased sensation and involuntary muscle spasms in his left foot.

The patient’s past medical history was significant for chronic back pain, and he was a former tobacco smoker with a 40 pack-year history. He denied any personal history or family history of connective tissue disease. Vital signs were: temperature 36.6 C, blood pressure 150/101 mmHg, heart rate 67 beats/min, respirations 18 breaths/min, SpO2 95%, body mass index (BMI) 30.1 kg/m^2^. The patient appeared oriented and in no apparent distress. On cardiovascular exam, the patient had a normal rate, regular sinus rhythm, and heart sounds were regular—no murmurs, rubs, or gallops. The patient’s femoral, popliteal, dorsalis pedis, and posterior tibial pulses were intact bilaterally.

**Figure 1:**
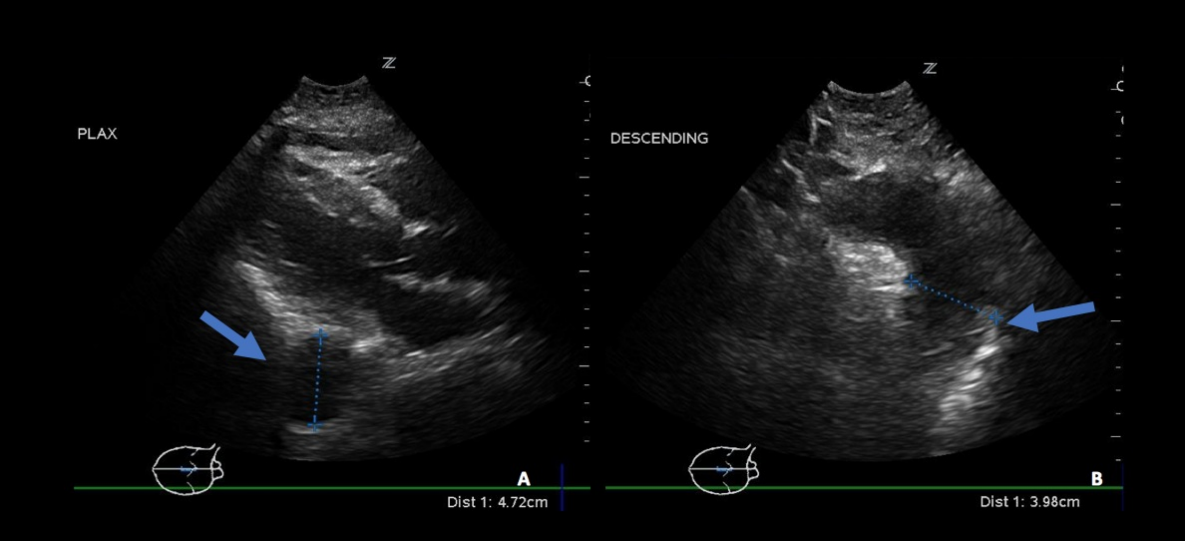
Thoracic aortic aneurysm. (A) Parasternal long axis (PLAX) imaging of the heart demonstrates an enlarged descending thoracic aorta. (B) Suprasternal Imaging demonstrates a grossly enlarged descending thoracic aortic root.

**Figure 2:**
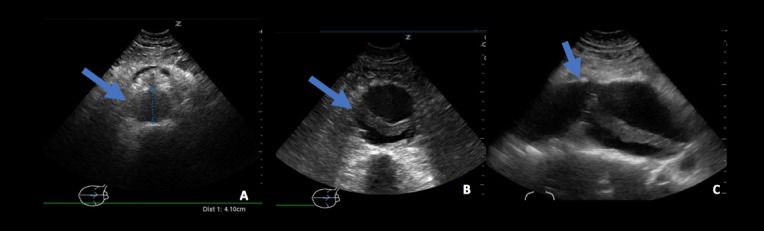
Abdominal aorta. (A) Transverse imaging of proximal abdominal aorta with aneurysm. (B) Transverse imaging of mid abdominal aortic aneurysm with intramural thrombus. (C) Sagittal imaging of mid abdominal aorta with intramural thrombus.

Given the patient’s complaint of tearing chest pain and transient neurological events, the emergency physicians (EPs) suspected aortic pathology. Given our patient’s transient paralysis and weakness of the lower extremities, a concern for transient ischemic attack (TIA) could not be ruled out. As a result, the EP began the diagnostic workup by performing a point of care ultrasound (POCUS) of the chest and abdomen while waiting for computed tomography (CT) imaging. POCUS demonstrated a normal thoracic aortic outflow track, dilated descending thoracic aortic aneurysm, and a dilated infrarenal AAA with an intramural thrombosis extending into the left common iliac artery (Figure 1 and Figure 2) (Video 1 and Video 2). The EP consulted vascular surgery and initiated transfer of the patient to a tertiary care facility as a result of the POCUS.

**Video 1:** Ultrasound of aneurysm with intramural thrombus. Transverse imaging of the mid abdominal aorta with intramural thrombus.

**Video 2:** Transverse distal abdominal aorta. Distal abdominal aortic aneurysm extending to iliac arteries. Thrombus of the left iliac artery is visualized at the end of the video.

**Figure 3:**
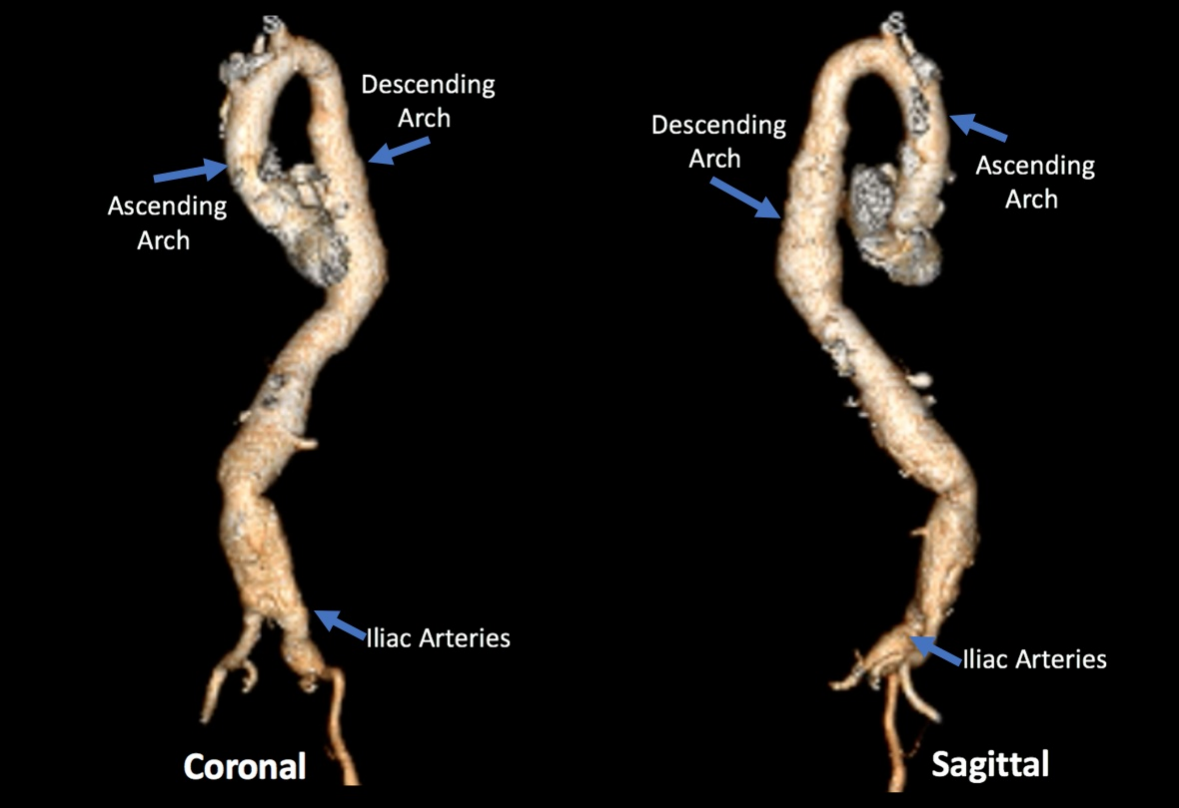
3-dimension aorta. Computed tomography of the aorta with 3-dimmensional reconstruction. Coronal and sagittal imaging of the thoracic and abdominal aorta.

**Figure 4:**
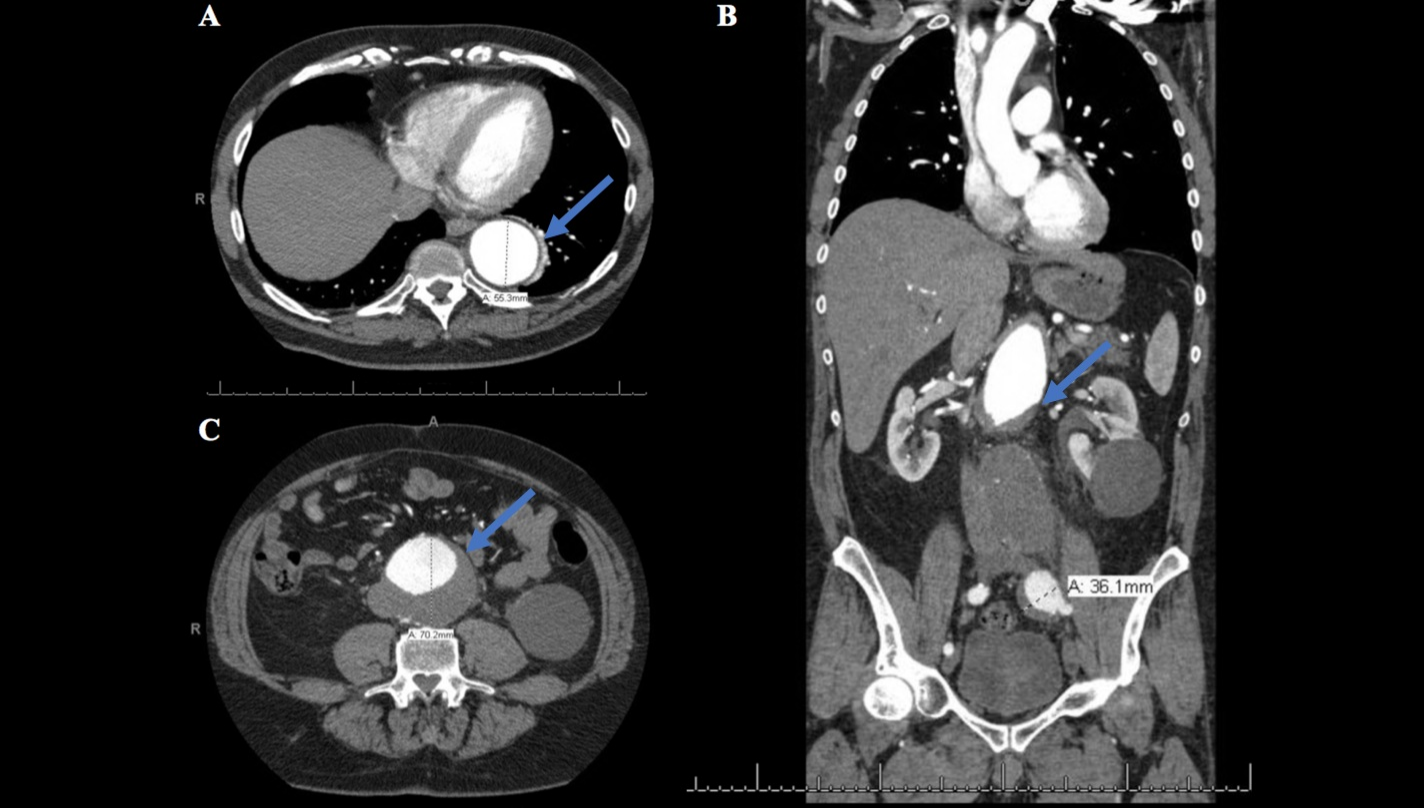
Computed tomography (CT). Transverse and coronal CT imaging of the thorax and abdomen with contrast which demonstrated an acute intramural hematoma extending from the ascending aorta, aortic arch, into the descending thoracic aorta. The aortic root measures up to 3.4 cm. The descending thoracic aorta is dilated and measures up to 5.5 cm. There is dilatation of the intra-abdominal aorta. At the level of the renal arteries, the aorta measures 4.8 cm. Infrarenally, there is an abdominal aortic aneurysm measuring up to 7 cm with eccentric thrombus extending over a length of 10.4 cm extending into the left iliac artery. (A) The descending aorta measured up to 5.5 cm. (B) The left iliac artery measured up to 3.6 cm. (C) The infrarenal aorta measured up to 7 cm.

While awaiting CT imaging, the patient was started on a nicardipine drip, hydralazine, and metoprolol for blood pressure control. CT imaging demonstrated the following: Acute intramural hematoma extending from the ascending aorta, aortic arch, into the descending thoracic aorta. The aortic root measures up to 3.4 cm. The descending thoracic aorta is dilated and measures up to 5.5 cm. There is dilatation of the intra-abdominal aorta. At the level of the renal arteries, the aorta measures 4.8 cm. Infrarenally, there is an AAA measuring up to 7 cm with eccentric thrombus extending over a length of 10.4 cm into the left iliac artery (Figure 3 and Figure 4).

## Discussion

AAA measuring 7 cm or larger have a 20-40% risk of rupture, and progression to aortic dissection accounts for 80% of all deaths in patients with aortic aneurysms [1, 3]. Aortic dissection should be suspected in any patient presenting with chest pain and elevated blood pressure. In this case, the clinicians suspected aortic pathology and their use of point of care ultrasound led to decreased time to consultation and transfer. This patient was found to have a type III thoracoabdominal aortic aneurysm with extensions from the ascending aorta to the infrarenal aorta and left common iliac artery.

In the literature, presentation of a widespread aortic aneurysm and temporary paralysis of the lower extremities is rare. In fact, most cases of aortic aneurysm or dissection only describe paralysis due to spinal cord ischemia, and include symptoms of urinary retention and bilateral lower extremity paralysis [4-8]. In this case, the distal abdominal aorta was found to have an intramural thrombus and a partially thrombosed left common iliac artery. These findings are consistent with the patient’s transient numbness and paralysis of his lower extremities.

POCUS has been successfully integrated into the curriculum of all emergency medicine (EM) residencies and all newly graduating EM physicians have clinical exposure to this imaging modality [9]. Ultrasound evaluation of AAA is uncomplicated and has been shown to easily integrate into an EPs skillset [10]. A recent systematic review and meta-analysis has demonstrated that EP use of bedside ultrasound for the diagnosis of AAA has a sensitivity of 99% and a specificity of 98% [10]. In this case, the EP was able to diagnose, consult, transfer, and treat the patient within 60 minutes of presentation.

## Conclusions

Our case illustrates the efficient use of ultrasound in the ED for the diagnosis and treatment of a unique presentation of an aortic aneurysm. Clinicians should be aware of the unique presentation of aortic aneurysms and dissection as well as the utility of ultrasound in the diagnosis of such pathology.
